# The Greenwood function shows close alignment with pitch perceived by cochlear implant patients with long, flexible electrode arrays and fine-structure stimulation

**DOI:** 10.3389/fnins.2025.1624499

**Published:** 2025-09-02

**Authors:** Andreas Büchner, Tobias Weller, Richard T. Penninger, Luke Helpard, Hanif M. Ladak, Sumit Agrawal, Thomas Lenarz, Daniel Schurzig

**Affiliations:** ^1^Department of Otorhinolaryngology, Hannover Medical School, Hannover, Germany; ^2^German Hearing Center (DHZ), Hannover, Germany; ^3^MED-EL, Innsbruck, Austria; ^4^Department of Otolaryngology-Head and Neck Surgery, Western University, London, ON, Canada; ^5^Department of Medical Biophysics, Western University, London, ON, Canada; ^6^Department of Electrical and Computer Engineering, Western University, London, ON, Canada

**Keywords:** tonotopy, mismatch, pitch matching, speech perception, music appreciation

## Abstract

**Introduction:**

The natural, tonotopic frequency distribution of the inner ear is typically described by the Greenwood function, which logarithmically projects the audible frequency spectrum onto the intracochlear basilar membrane. Recent developments in cochlear implant (CI) programming aim to improve sound quality and music perception through consideration of the frequency distribution as described by the Greenwood function when assigning frequency bands to the individual contacts of the electrode array. This approach is commonly referred to as anatomy-based fitting (ABF). However, empirical validation of the Greenwood function to accurately describe pitch as perceived by CI users is lacking.

**Methods:**

Twelve CI patients with single-sided deafness (SSD) participated in the study. A pitch matching task was conducted at four different appointments and with two different fitting maps (standard and ABF). At each test appointment, participants were asked to set the frequency of a pure tone presented through a loudspeaker to the pitch perceived when stimulated with the single contacts of the CI electrode array. The cochlear anatomy of the patients was reconstructed based on clinical imaging to derive the location of the stimulating contacts relative to the basilar membrane, allowing for the comparison of the pitch perceived by the patients to the frequency suggested by the Greenwood function for each stimulating contact.

**Results:**

In general, subjective pitch percepts were found to agree well with the frequency suggested by the Greenwood function independent of subject, contact, or applied fitting map. Differences between pitch matches and Greenwood were found to be not statistically significant. At least part of the outcomes of previous studies reporting a basal frequency shift can be explained by the tonotopic mapping functions applied within these studies.

**Discussion:**

The present results suggest that the Greenwood function is well-suited for representing the tonotopic frequency distribution not only for normal hearing subjects but for CI recipients as well. Further advances in frequency mapping should also take the neural health of the cochlea into account, allowing for additional individualization of frequency mapping in CIs.

## 1 Introduction

Cochlear implants (CIs) have revolutionized the treatment for individuals with severe-to-profound hearing loss, offering a functional restoration of hearing capabilities (Clark, 2003; Lenarz, 2017). These sophisticated devices consist of an externally worn sound processor and an internal implant connected to an electrode array, typically comprising between 12–22 electrode contacts (Dhanasingh and Jolly, 2017). The array is positioned within the scala tympani to electrically stimulate neural elements in the cochlear modiolus. The external sound processor plays a crucial role, decomposing incoming audio signals into distinct frequency bands. It then determines the amplitude—and in some systems also the frequency—of signals within these bands, with each band corresponding to a specific intra-cochlear electrode contact. Electrical currents, proportional to these band-pass amplitudes, are then delivered to the respective contacts for auditory perception. In some systems, the frequency of the signal in such a channel is also transmitted by controlling stimulation rate accordingly. In natural hearing, the human cochlea employs a tonotopic organization along the basilar membrane, where sound frequencies ranging from approximately 20–20,000 Hz are logarithmically mapped. It is anticipated that a person with normal hearing can discern 1,400–1,600 different frequencies (Shower and Biddulph, 1931), a level of precision that presents a significant challenge for CI technology to match. This frequency mapping is elegantly described by the Greenwood function (Greenwood, 1990), which illustrates the spatial arrangement of 3,500 inner hair cells along the cochlear length (Pickles, 2013), tuned systematically to frequencies, *f,* from high (base) to low (apex):


(1) $$f=A(10^{\mathrm{ax}}-k)$$

For human frequency perception, the coefficients of this function were defined as *A* = 165.4, *a* = 2.1 and *k* = 0.88, which yields a projection of the abovementioned frequency spectrum onto the entire length of the basilar membrane (BM). The parameter *x* within the Greenwood function describes the relative length along the BM from apex (*x* = 0) to base (*x* = 1). It is important to note that the Greenwood function is merely a mapping function projecting the audible frequency range of 20 Hz to 2000 Hz onto the BM, a structure which is not visible in clinical imaging. Clinical application of the Greenwood function hence requires models to approximate the exact position of the BM based on anatomical structures which can be clearly distinguished in clinical imaging, as proposed by Boëx et al. (2006), Stakhovskaya et al. (2007) and Helpard et al. (2021b).

Despite significant advancements in CI technology, users often encounter substantial challenges in complex listening environments, such as those with background noise or reverberation, or when listening to music. It is clear that higher-order processing stages play a crucial role in speech understanding (Collison et al., 2004; Rönnberg et al., 2013; Finke et al., 2016). However, a set of peripheral factors at the electrode-nerve-interface also contribute to the observed variability in challenging acoustic scenarios, including the intracochlear electrode placement (Holden et al., 2013), channel interactions (Boëx et al., 2003; Bierer, 2010) which lead to poor frequency resolution, and the small electrical dynamic range (de Graaff et al., 2020). Electrode placement and the coverage of the cochlea achieved by the electrode determines the accurate rendering of pitch, a fundamental auditory percept. Pitch carries essential information, extending beyond the realm of music perception to include nuances in language processing, such as speaker identity, prosody, and lexical tones. Moreover, the challenge of pitch perception in CI users is further nuanced in cases of single-sided deafness (SSD). In such scenarios, individuals have the unique ability to compare the pitch perceived through the CI in one ear with that heard naturally in the other. This comparison offers invaluable insights into the potential disparities in pitch perception between the implanted and non-implanted ears, shedding light on the limitations and possible areas of improvement for CI technology in replicating the complex nature of auditory perception. Unfortunately, previous pitch matching studies on SSD subjects revealed controversial results: while some studies demonstrated close alignment of perceived pitch with the Greenwood function (Vermeire et al., 2008; Adel et al., 2019), others showed a basal shift of the perceived frequencies with respect to Greenwood (Peters et al., 2016; Dorman et al., 2007; Boëx et al., 2003; Zeng et al., 2014). First investigations trying to explain these differences could show that type of stimulation can influence the perceived pitch (Adel et al., 2019), which may account for some of the outcome variability. In addition, different models were applied to employ Greenwood’s frequency distribution, which may in itself yield differences in tonotopic mapping and account for some of the discrepancies.

In the pursuit of enhancing CI users’ experiences, especially in challenging listening conditions, ongoing research focuses on refining the technology to more closely mimic natural hearing mechanisms. This includes improving the CI’s ability to process pitch information accurately, an endeavour critical for a more natural and satisfying auditory experience. A prerequisite for natural stimulation is sufficient coverage of the intracochlear neural structures by the CI (Li et al., 2020; Li et al., 2021; Helpard et al., 2021b), which is dependent on both cochlear size (Erixon et al., 2009; Erixon and Rask-Andersen, 2013; Meng et al., 2016; Würfel et al., 2014) and CI electrode length (Timm et al., 2018; Dhanasingh and Jolly, 2017). Sufficient cochlear coverage also minimizes the mismatch between natural frequency perception and the frequency bands assigned to the individual CI electrode contacts (Landsberger et al., 2015). Recent results have demonstrated that deep CI electrode insertions beyond 540° result in a smaller mismatch between the cochlea’s tonotopic frequency distribution and the standard frequency assignment of a CI (Canfarotta et al., 2020), which might explain the large number of studies correlating improved speech perception with deeper CI electrode insertions (Weller et al., 2023; Canfarotta et al., 2022; Breitsprecher et al., 2023). However, pitch perception in CI users is still moderate (Zeng et al., 2008), which is why advanced fitting strategies were introduced to align the CI’s frequency assignment more closely to a patient’s natural frequency distribution (Alahmadi et al., 2024). First investigations of this new anatomy-based fitting (ABF) concept showed significant improvements in speech perception (Kurz et al., 2022; Kurz et al., 2023; Dillon et al., 2023).

While technological advances have been employed to study interindividual differences of the intracochlear neural structures targeted by CI stimulation in extraordinary detail (Elfarnawany et al., 2017; Li et al., 2020), the translation of anatomical/geometrical information to frequency is currently always conducted using Greenwood’s approach (Greenwood, 1990) or derivatives thereof (Stakhovskaya et al., 2007; Helpard et al., 2021b). It is important to note, however, that Greenwood employed various assumptions to derive his formula. Furthermore, there is a lack of evidence that Greenwood’s function sufficiently describes the tonotopic organisation of the electrically stimulated cochlea. This could explain some of the variations in outcomes with ABF-based CI fitting strategies. The aim of the present study was to explore whether the Greenwood formula accurately describes the projection of the audible frequency spectrum onto the intracochlear neurons when electrically stimulated via a CI electrode.

## 2 Materials and methods

### 2.1 Ethics statement

The study was approved by the ethical review board of the Hannover Medical School under review number 8445 and was conducted in accordance with the Declaration of Helsinki. All participants provided written consent prior to the first study appointment and received reimbursement of cost of travel associated with participation in the study.

### 2.2 Patient cohort

Initially, 13 subjects (seven female, six male) were recruited from the CI database at the German Hearing Center (DHZ) and enrolled in the study. One subject dropped out due to personal reasons related to the COVID-19 pandemic, resulting in a total study cohort of 12 subjects (7 female, 5 male). All subjects were CI users with postlingual single-sided deafness (SSD), native German speakers, and older than 18 years (mean: 52.9 years; range: 35.5–80.6 years) at the time of enrolment in the study. Subjects were only enrolled in the study if they had no functional residual hearing on the implanted side (ipsilateral hearing threshold of ≥ 65 dB HL at 250 Hz), and normal hearing or near-normal hearing on the non-implanted side (contralateral four-frequency pure-tone average, 4-PTA, of ≤ 30 dB HL). Additionally, subjects were only included in the study if they had at least 6 months experience with their implant (mean: 20.2 months; range: 8.6–52.0 months), and if they had at least one prior test result greater than 0% correct in the Hochmair, Schulz, Moser (HSM) sentence test in noise at 10 dB SNR (Hochmair-Desoyer et al., 1997). Of the 12 subjects, six were implanted with the Synchrony and six with the Synchrony 2 implant (MED-EL, Innsbruck, Austria), with either the FLEX28 electrode array (*n* = 11) or the FLEXSOFT electrode array (*n* = 1). Subjects were using either the RONDO 2 (*n* = 5), the SONNET (*n* = 3), or the SONNET 2 (*n* = 4) audio processors prior to the study. Due to technical limitations of the SONNET and the RONDO 2 audio processors, subjects using these processors were fitted with a SONNET 2 audio processor for the duration of the study. A detailed overview of the subjects’ demographic data is given in Table 1. Note that all study subjects were fitted with the FS4 stimulation strategy. The stimulation rates stated in Table 1 hence do not refer to the apical channels which also deliver temporal information when defined as fine-structure channels during fitting. In case of the present study, this was the case for the apical 4 contacts for all subjects.

**Table 1:** Demographic data of the present study cohort (*n* = 12).

ID	Etiology	Language status	Duration of deafness [yrs]	Age at study [yrs]	Experience with CI [yrs]	Implant	Audio processor	Electrode array	Contralateral PTA [dB HL]	Cochlear angle [°]	Insertion angle [°]	Stimulation rate std. map [pps]	Stimulation rate ABF map [pps]
subj01	SSNHL	Postlingual	2.6	37.4	1.1	Synchrony	Rondo2	FLEX28	5	959	573	1,263	1,237
subj02	SSNHL	Postlingual	5.9	56.3	0.7	Synchrony 2	Sonnet2	FLEX28	20	923	550	1,230	1,261
subj03	SSNHL	Postlingual	0.4	35.5	1.0	Synchrony 2	Rondo2	FLEX28	5	957	550	1,351	1,217
subj04	SSNHL	Postlingual	6.9	61.1	1.8	Synchrony	Sonnet	FLEX28	21.25	808	515	1,299	1,299
subj05	SSNHL	Postlingual	0.6	56.0	2.6	Synchrony	Sonnet	FLEX28	6.25	837	525	1,345	1,299
subj06	SSNHL	Postlingual	1.6	55.8	1.2	Synchrony 2	Rondo2	FLEX28	17.5	821	629	1,250	1,250
subj07	SSNHL	Postlingual	2.6	51.8	2.7	Synchrony	Rondo2	FLEX28	11.25	885	570	1,266	1,266
subj08	SSNHL	Postlingual	0.5	40.8	2.1	Synchrony	Rondo2	FLEX28	8.75	896	586	1,261	1,237
subj09	SSNHL	Postlingual	12.2	80.6	4.3	Synchrony	Sonnet	FLEX28	23.75	870	521	1,282	1,299
subj10	Unknown	Postlingual	0.3	54.4	0.9	Synchrony 2	Sonnet2	FLEX28	12.5	870	590	1,304	1,304
subj11	Unknown	Postlingual	0.1	68.5	0.9	Synchrony 2	Sonnet2	FLEX28	13.75	863	571	1,261	1,261
subj12	SSNHL	Postlingual	0.9	36.1	0.9	Synchrony 2	Sonnet2	FLEXSOFT	15	856	637	1,242	1,242

Terminology: SSNHL, severe sensorineural hearing loss; CI, cochlear implant; PTA, pure-tone average; pps, pulse per second; ABF, anatomy-based fitting.

### 2.3 Anatomy-based fitting

**Figure 1:**
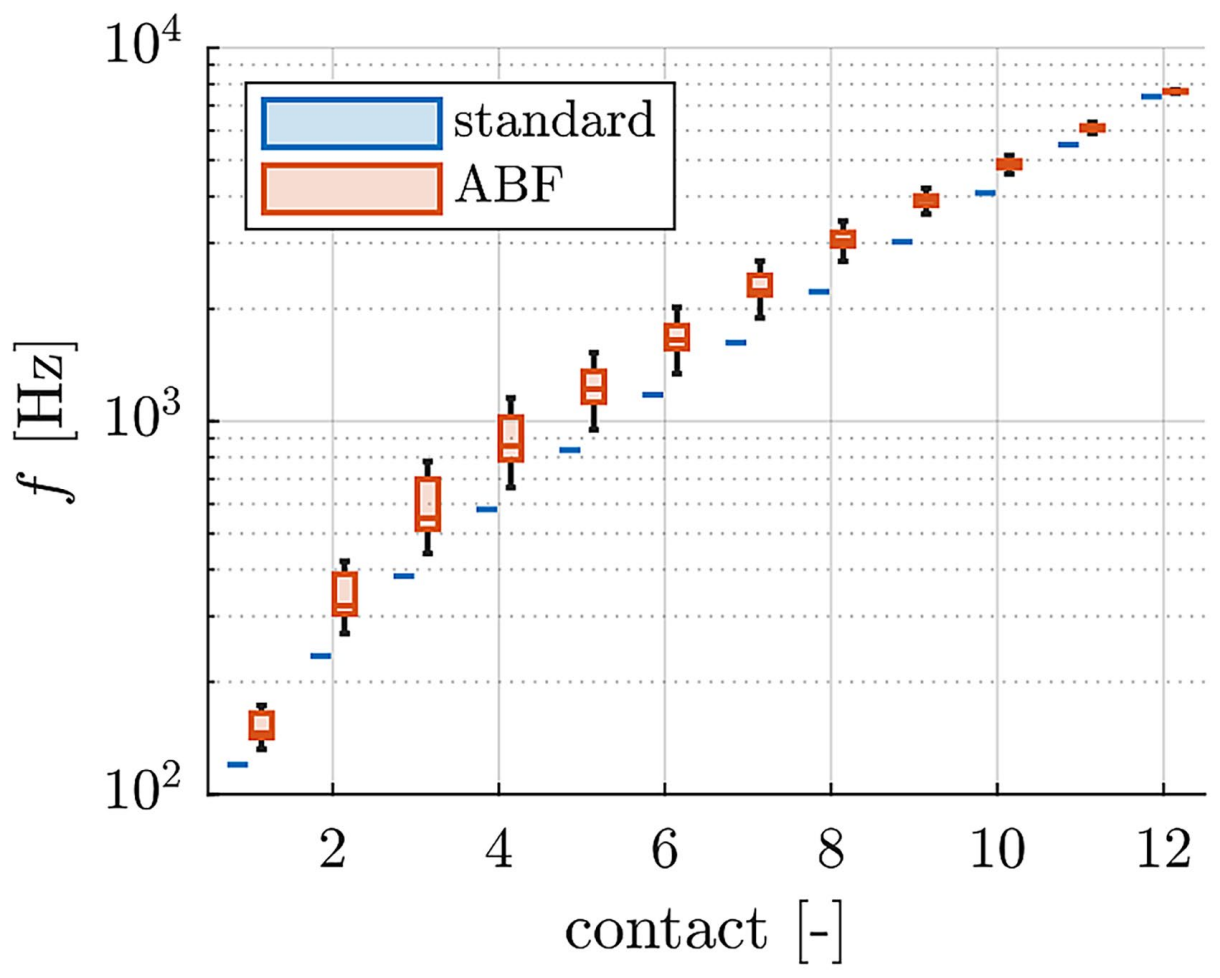
Comparison of center frequencies of standard and ABF maps of all study participants.

All computations were conducted with Matlab (version R2023a, MathWorks, Natick, MA, USA). The lateral wall (LW) and inserted CI electrode array were traced manually within each participant’s clinical cone-beam CT scan using Osirix MD (version 2.5.1 64bit, Pixmeo SARL, Switzerland) according to our previously reported protocol (Timm et al., 2018; Schurzig et al., 2018a, 2018b). In brief, the LW was traced by placing points along its contour from the center of the round window (RW) to the apex. Each CI array was segmented by placing the first point within the center of the RW, followed by one point within the center of each electrode contact artifact from base to apex. Using Matlab, spline curve interpolation and the HelReg registration method (Schurzig et al., 2018a) were applied to this geometrical data to reconstruct the path of the inserted CI array relative to the respective LW spiral. From the reconstructed path, the number of turns of the cochlea as well as the insertion angle of all electrode contacts could be determined. The LW spiral path was used to compute the individual, tonotopic frequency distribution of the respective organ of Corti (OC) and subsequently assign a tonotopic frequency to each electrode contact based on its location. The patient-specific OC frequency distributions for creating the AFB maps were computed using the function proposed by Helpard et al. (2021b), which employs the number of cochlear turns to tune the tonotopy to the individual patient. Finally, the ABF map itself was created by setting the center frequencies of frequency bands assigned to electrode contacts with tonotopic frequencies between 1–3 kHz to these tonotopic electrode frequencies (strictly tonotopic fitting) and distributing the center frequencies of the remaining electrode contacts logarithmically such that the entire frequency range of the audio processor between 70–8,500 Hz was utilized. An overview of both the standard and ABF maps of each participant is shown in Supplementary Figure S1. A summary of the center frequencies of standard and ABF maps of all participants is shown in Figure 1.

### 2.4 Study design and equipment

The purpose of the present study was to assess if the Greenwood function can be used to describe the place-frequency or place-pitch relationship of the electrically stimulated cochlea or, in other words, to assess whether the pitch that is created by an electrode contact corresponds to the frequency that is assigned to the intracochlear place of that electrode contact via the Greenwood function. In principle, this pitch should only be a function of electrode place and should not be related to the frequency band that is assigned to an electrode in the CI system. However, in order to rule out any such effect, two frequency assignments were investigated in the study.

**Figure 2:**
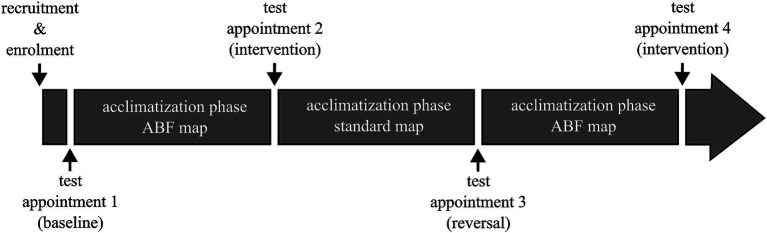
Schematic of the overall study design. Acclimatization phases were at least 4 weeks long. Pitch matching was conducted at each test appointment with both the standard and ABF map.

That is why the study utilized a repeated measures design, following an A-B-A-B pattern, resulting in a total of four study appointments for each subject spaced at least 4 weeks apart (Figure 2). In this pattern, “A” denotes a study interval during which subjects used the standard map, and ‘B’ denotes a study interval where the ABF map was used. Given that all participants were experienced CI users accustomed to the standard map, we bypassed the initial “A” interval. Subsequently, the first session only served to establish a baseline, assessing participants in their accustomed state with the standard map.

At the initial study appointment, contralateral hearing thresholds were assessed if the previous measurement was over 12 months old. Subsequently, using the MAESTRO CI fitting software (MED-EL) all programs except for the most frequently used program were removed from the subject’s audio processor. A second program was configured using the same Maximum Comfort Level (MCL) and Threshold (THR) settings of the primary program, but with the underlying frequency assignment switched to the newly generated ABF map. The stimulation rate for this program was set to the highest available, as detailed in Table 1.

For a brief period, subjects were allowed to switch between the two programs to ensure the new program was adequate before starting the pitch matching experiment. After completing the experiment, the original programs on the audio processor were restored, but with the underlying frequency assignment changed to the ABF map in all programs. This prevented subjects from reverting to their old map, though they were informed they could visit the clinic to adjust settings if the new configuration proved inadequate.

At the end of the second appointment, the map was reverted to the standard map and switched back again to the ABF map at the conclusion of the third appointment.

During the pitch matching experiment, subjects were seated in a sound-treated room. A loudspeaker was positioned 1 meter away from the subject, on their normal hearing side. The experimental setup included a laptop equipped with a U-Phoria UMC202HD USB audio interface (Behringer, Willich, Germany), which ran the PsyWorks software (MED-EL) for stimulus generation and presentation. The audio interface’s stereo output was configured such that one channel was connected to the loudspeaker and the other directly to the subject’s CI audio processor via an FM cable. The audio processor was running in live mode, employing the settings for the according study appointment mentioned above.

A second monitor was connected to the laptop and positioned in front of the subjects to display the PsyWorks graphical user interface (GUI). Subjects interacted with this interface using a mouse, enabling them to control the progression of the experiment independently. Throughout the experiment, the auditory stimuli consisted of sine tones. Triangular frequency modulation with a modulation frequency of 5 Hz and a modulation deviation of ±5% around the nominal frequency of the sinusoids was applied to avoid amplitude fluctuations due to standing waves during free-field presentation.

### 2.5 Pitch matching experiments

Before commencing the pitch matching experiment, a loudness balancing procedure was conducted for the stimuli (sinusoids) used throughout the pitch matching experiment. Initially, the loudness of stimuli at seven different frequencies (95 Hz, 180 Hz, 340 Hz, 630 Hz, 2,250 Hz, 4,200 Hz, and 8,000 Hz) was balanced against a reference stimulus of 1,200 Hz at 65 dB SPL. This was done on the normal hearing side, with all sounds presented through the loudspeaker. Subsequently, the already adjusted stimuli were balanced with their corresponding frequencies presented over the CI, including the 1,200 Hz stimulus.

During this procedure, subjects-initiated playback of the reference stimulus, which automatically triggered the presentation of the comparison stimulus. They could then adjust the loudness of the second stimulus using one of four buttons to increase or decrease the volume in small (1 dB) or large (3 dB) increments. After each playback, subjects had the option to make further adjustments, repeat the process, or proceed to the next stimulus once they perceived both stimuli as equally loud. Upon completing all adjustments for the seven frequencies, the settings were saved and applied to the stimuli for the second part of the loudness balancing.

In the pitch matching experiments, the reference stimulus was always presented via the CI, followed by a second stimulus played through the loudspeaker. Subjects adjusted the frequency of the second stimulus using a vertical slider in the graphical user interface (GUI), which operated on a logarithmic scale. This allowed for frequency adjustments between 100 Hz and 7,950 Hz. Adjustments were made by either using the arrow buttons at the top and bottom of the slider for minor changes, clicking directly on the slider bar for larger shifts, or dragging the slider to the desired position.

To ensure consistent loudness across varying frequencies, the PsyWorks software automatically adjusted the presentation level for each chosen frequency by interpolating between the previously measured level adjustments from the loudness balancing procedure. Pitch matching was assessed either with the standard map active and stimulus frequencies set to the center frequencies of the standard map, or with the ABF map active and stimulus frequencies set to the center frequencies of the ABF map. The stimuli were presented to the CI ear via direct-in to an audio processor running the FS4 coding strategy and with the audio processor microphone muted. Stimuli to the normal hearing ear were presented via the loudspeaker.

Note that the experiments were only conducted for the uneven electrode contacts to keep the time span of the experiments at a reasonable level. Furthermore, subjects were allowed breaks as needed during the sessions. Each condition was measured three times, with the initial frequency of the second stimulus set to either the lowest (100 Hz), highest (7,950 Hz), or a mid-range frequency within the available range for the first presentation. In each run, the presentation order of the different stimulus frequencies was randomized. The presentation order of the runs (starting frequency bottom, center, or top) and the presentation order of the conditions were pseudo-randomized between subjects and remained the same for all four appointments of the study. Overall, each subject completed 144 pitch matching trials, distributed across four appointments with two maps, three runs each, and six frequencies per run.

### 2.6 Quantification of pitch matching ability

The pitch matching ability of individual subjects was rated by quantifying the spread of pitch matches for an individual contact. In a first step, all pitch matches *f*_PM_ derived for a specific contact were transformed into deviations Δ*f*_PM_ from the mean of the respective values ${\bar{f}}_{\mathrm{PM}}$ of that contact, stated in semitones, according to Equation 2:


(2) $$\Delta f_{\mathrm{PM}}=12\;\log_{2}(\frac{f_{\mathrm{PM}}}{{\bar{f}}_{\mathrm{PM}}})$$

Based on the resulting *N* frequency deviation values $\Delta f_{\mathrm{PM}}$ , the total root-mean-square deviation for an individual subject was computed as stated in Equation 3:


(3) $$\Delta f_{\mathrm{RMS}}=\sqrt{\frac{1}{N}\sum_{i=1}^{N}{\left(\Delta f_{\mathrm{PM},i}\right)}^{2}}$$

i.e., smaller values of $\Delta f_{\mathrm{RMS}}$ correspond to more consistent pitch matches and thus better pitch matching ability.

### 2.7 Greenwood reference

**Figure 3:**
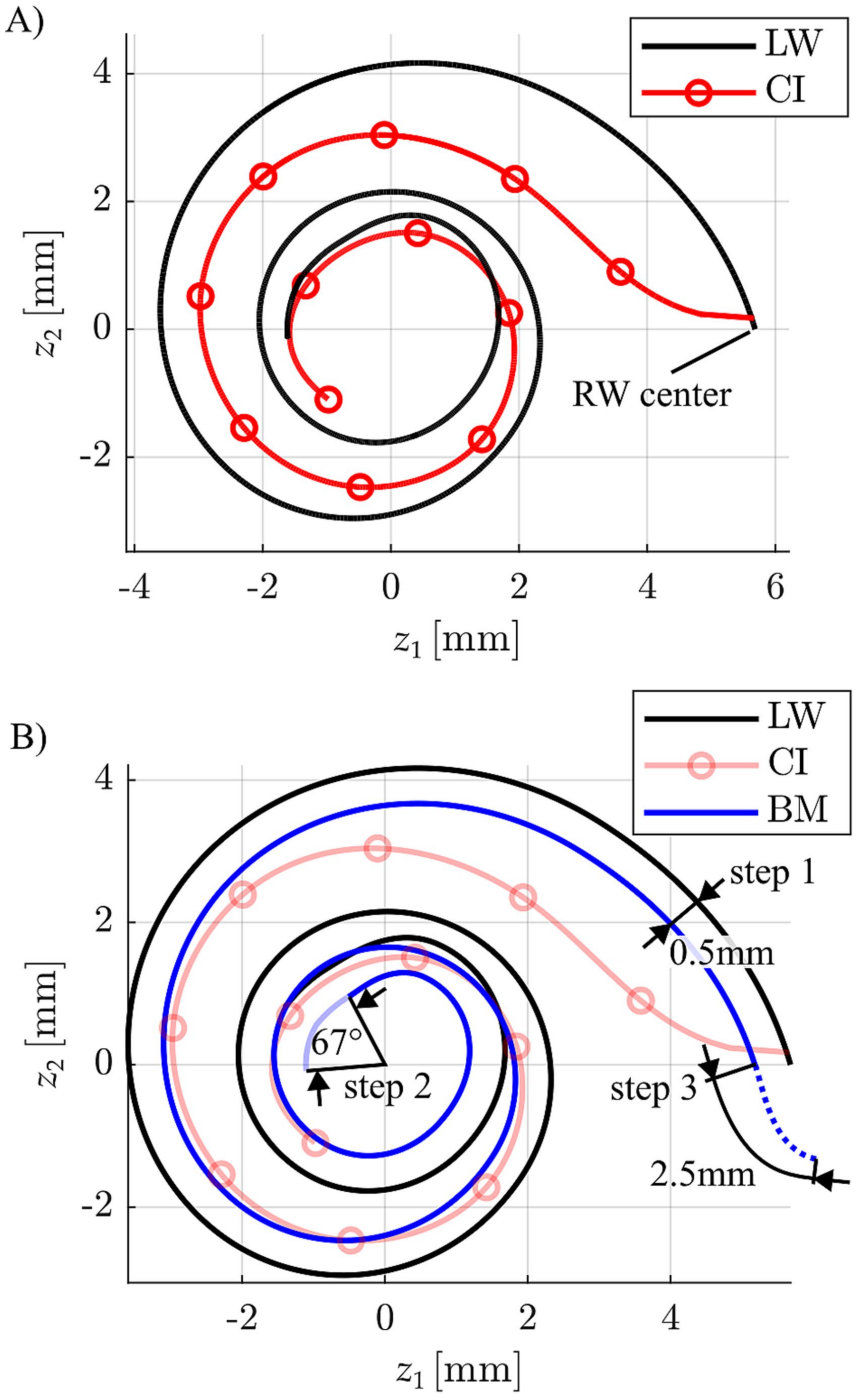
**(A)** Depiction of a CI array reconstruction (circles indicate the individual stimulation contact locations) relative to the respective lateral wall (LW) spiral. **(B)** Approximation of a basilar membrane (BM) profile based on the corresponding LW by a 0.5 mm projection of the LW toward the modiolar axis, an apical reduction of 67° to account for the helicotrema and a basal 2.5 mm extension for consideration of the hook region length of the BM.

Anatomically speaking, the focus of this investigation was the basilar membrane (BM), as the Greenwood function maps the auditory frequency range from 20–20,000 Hz onto this structure (or rather the OC on top of the BM). Each individual BM was hence reconstructed based on the anatomical information available from clinical imaging (cf. Section 2.3). A visual representation (top-down view) of this three-dimensional reconstruction technique is shown in Figure 3.

Firstly, the LW profile was projected medially towards the modiolus by 0.5 mm, aligning with the average distance between the LW and BM found in previous anatomical studies (Alexiades et al., 2015; Helpard et al., 2021b). This projection of the LW was then extended basally by 2.5 mm, which corresponds to the average length of the BM within the cochlear hook region (Helpard et al., 2021a), and shortened apically by 67° reflecting the average angular range of the helicotrema (Helpard et al., 2020). It is important to note that while our BM reconstruction incorporates key anatomical findings from the research group around Helpard et al.—whose use of Synchrotron radiation Phase-Contrast Imaging has yielded the most detailed cochlear imaging data currently available (Elfarnawany et al., 2017)—we did not adopt their mathematical modelling approach for creating the Greenwood frequency reference, but only to create the ABF maps within the present study (cf. Section 2.3). Specifically, Helpard et al. proposed a sinusoidal function to describe angle-to-frequency relationships based on their anatomical data. In contrast, our method employs a purely numerical reconstruction strategy, which avoids the need for simplifying assumptions inherent in fitting mathematical models to anatomical structures. By directly integrating key findings from high-resolution anatomical data, we believe our approach offers a more accurate and anatomically faithful representation of the BM. Accordingly, the Greenwood function was applied to this numerically reconstructed BM to serve as the reference for all subsequent analyses.

Secondly, stimulation sites along the BM were defined based on the reconstructions of the CI array and BM. The closest location along the BM was computed for each CI contact and converted into relative BM length *x,* according to the Greenwood function (Greenwood, 1990).

## 3 Results

The initial step of the pitch matching analysis focused on the derivation of factors influencing the pitch matching responses given by the subjects. This was done by conducting an n-way analysis of variance (ANOVA) using Matlab’s build-in function *anovan*, providing the single pitch matches *f*_PM_ of all subjects as the dependent variable and the factors “subject” (1–12), “contact” (1,3,5,7,9,11), “map” (standard or ABF) and “study interval” (A, standard map or B, ABF map; cf. Section 2.4) as independent variables. The analysis yielded that the factors “subject” (*p* < 0.001) and “contact” (*p* < 0.001) significantly affected pitch matches. As suspected (cf. Section 2.4), the factors “map” (*p* = 0.586) and “study interval” (*p* = 0.247) did not. However, it should be mentioned that a post-hoc power analysis revealed that the power to detect a small effect (η^2^ = 0.01) was only 17, and 71% for a medium effect (η^2^ = 0.06). Only large effects (η^2^ = 0.14) could be detected with high power (98%).

**Figure 4:**
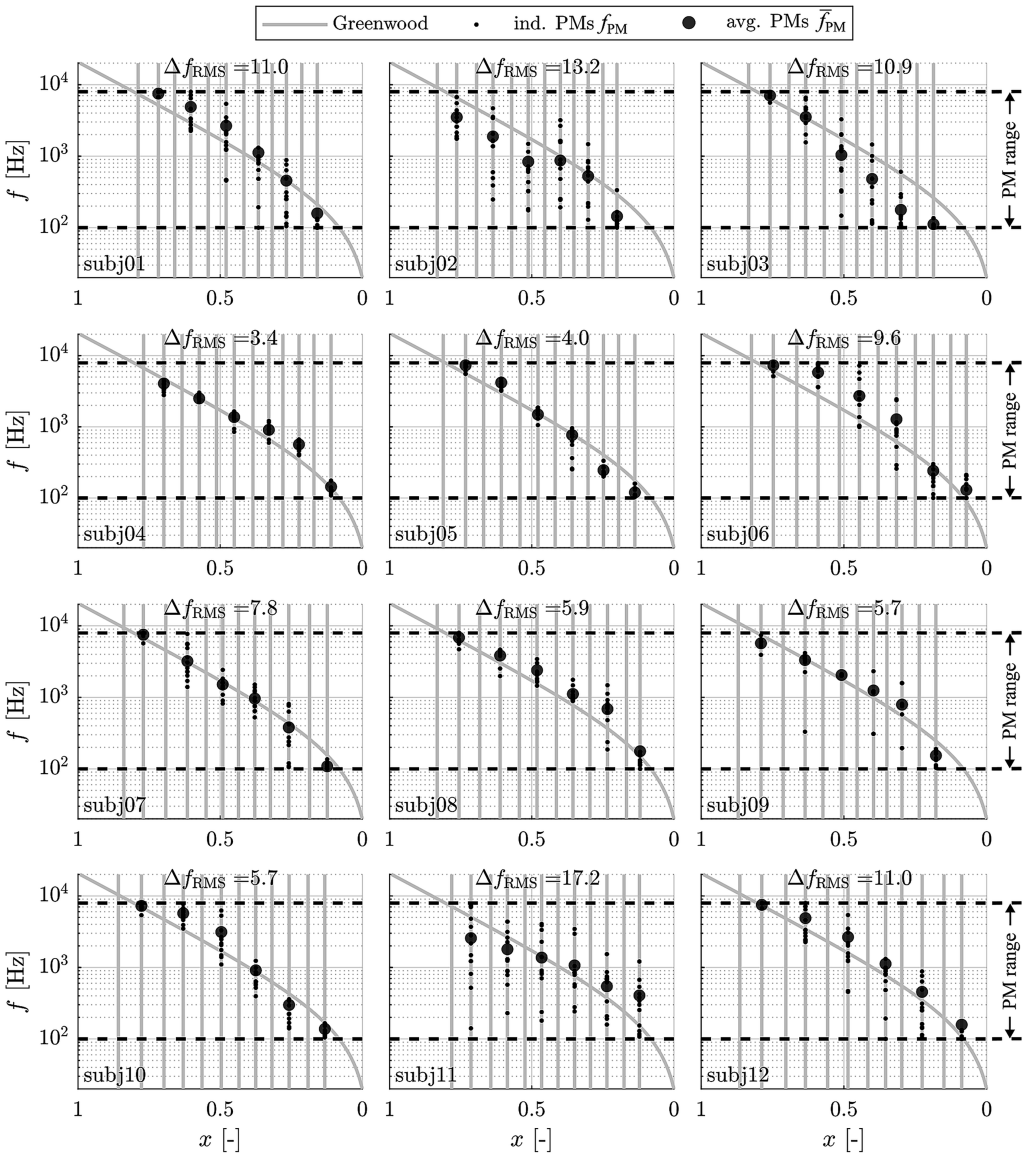
Perceived frequencies (small dots: individual matches *f*_PM_; large dots: average values ${\bar{f}}_{\mathrm{PM}}$ ) in response to contact specific stimulation (at contacts 1, 3, 5, 7, 9 and 11) with the respective center frequencies, pooled across maps, appointments and familiarization periods and displayed along the relative BM length *x*. Vertical grey lines represent the individual contact locations. The dashed lines represent the range of possible responses *f*_PM_ from 100–7,950 Hz.

Pitch matches were then pooled across maps and study intervals for each subject and electrode contact, respectively. The corresponding results are shown in Figure 4, where individual pitch matches *f*_PM_ (small dots) and mean values ${\bar{f}}_{\mathrm{PM}}$ (larger dots) are depicted over the relative BM length *x* value closest to the stimulating contact for each subject. The locations (i.e., *x* values) of the CI contacts with respect to the BM are indicated as vertical grey lines. Since contact specific stimulation was only conducted for every other contact starting at the most apical one, frequency matches were only available for every other contact as well. All responses were pooled across maps and study intervals since these factors did not have a significant effect on perceived pitch. The standard Greenwood function is provided as a reference. Individual pitch matching ability scores, Δ*f_RMS_*, are stated for each subject as well.

The depicted results clearly demonstrate that subjects varied in their ability to perform the pitch matching task in a reproducible manner, as indicated by Δ*f*_RMS_ values ranging from 3.4–17.2 semitones (i.e., up to almost one and a half octaves). The results also show that pitch matches are generally in good agreement with the Greenwood function, whereby for subjects with lower Δ*f*_RMS_ values, or with more consistent pitch matches, the pitch matches were generally closer to Greenwood than for subjects with higher Δ*f*_RMS_ values. Due to the large range of Δ*f*_RMS_ values and the corresponding substantial variation in capability to perform the pitch matching task, all subsequent analyses were performed for both the full study cohort (12 subjects) and for good performers only. Good performers were defined as subjects who showed a Δ*f*_RMS_ of less than 6 semitones, i.e., half an octave (5 subjects).

The results depicted in Figure 4 also show that for the most apical contact, 7 of the 12 subjects responded with the lowest possible frequency (100 Hz) in some cases (14% of all matches; 2% of matches by good performers). The same holds true for responses to the most basal stimulation, where the highest possible frequency (7,950 Hz) was set in some cases (27% of all matches; 11% of matches by good performers), indicating that these subjects might have responded with values beyond the range of 100–7,950 Hz to truly match their subjective percept.

**Figure 5:**
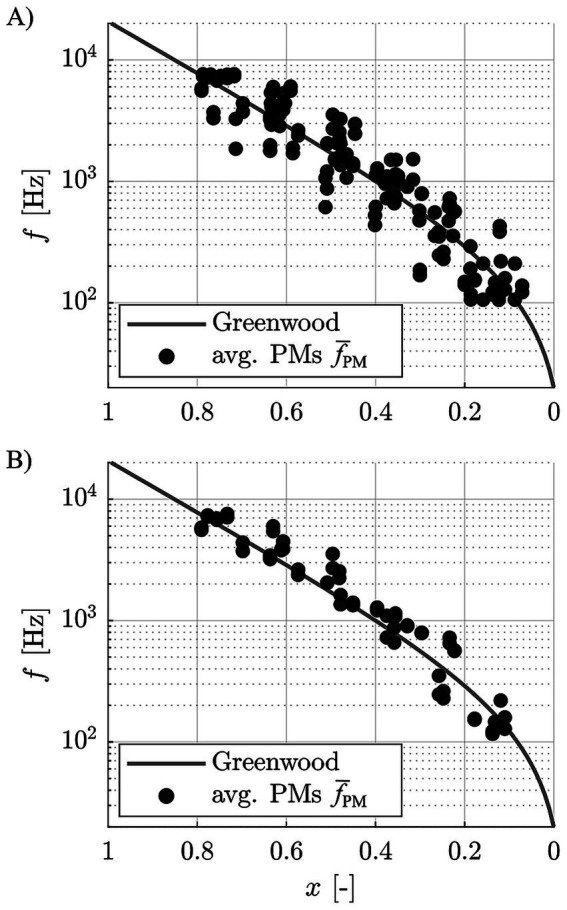
Pooled, contact specific average pitch matches ${\bar{f}}_{\mathrm{PM}}$ of **(A)** all subjects, and **(B)** the good performers only, relative to the Greenwood function.

Figure 5 shows the aggregate data (average PMs ${\bar{f}}_{\mathrm{PM}}$ ) both for the entire group and the good performers alone relative to the Greenwood function, again showing a good agreement of the subjects’ perceived frequencies to the function.

**Figure 6:**
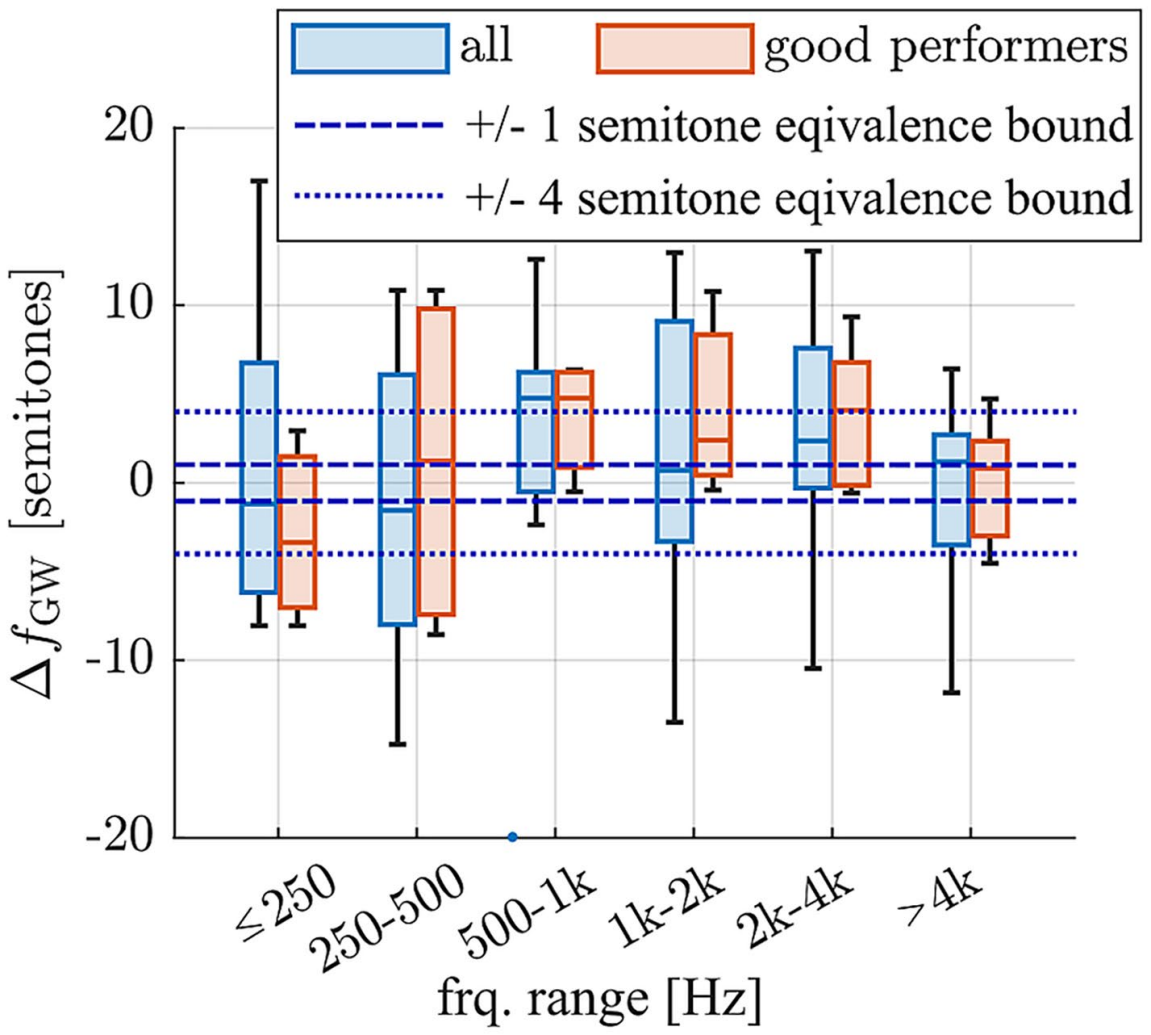
Deviation of average pitch matches ${\bar{f}}_{\mathrm{PM}}$ of each subject and contact from the frequency expected based on the Greenwood function, divided into frequency ranges based on the expected Greenwood frequencies and displayed for the full cohort (*n* = 12 subjects) and the sub-cohort of good performers which provided reproducible pitch matching results (*n* = 5 subjects).

To quantify the deviation of pitch matches from the Greenwood function, the difference between the contact specific, average frequencies ${\bar{f}}_{\mathrm{PM}}$ , and the respective Greenwood values was calculated in semitones for each subject. The Greenwood frequency of each contact was employed to then group these deviations into frequency ranges around usual audiometric testing frequencies, which is depicted in Figure 6. The Wilcoxon Signed Rank Test was performed to evaluate if these grouped differences were significantly different from 0, which would entail statistically significant differences of the derived pitch matches to the Greenwood function. As shown in Supplementary Table S1, none of the differences were found to be statistically significant. This suggests that the perceived frequencies closely align with those proposed by Greenwood, with no significant differences observed for either the entire group or the subset of good performers. In addition, we tested for equivalence of the pitch matches with the Greenwood function using a two one-sided test (TOST) with the narrow equivalence interval of ± 1 semitone, which is often difficult to distinguish even for normal hearing subjects (Jiang et al., 2013; McClaskey, 2013). The TOST yielded *p*-values of *p* = 0.021 (lower bound) and *p* = 0.418 (upper bound) for all participants and *p* = 0.004 (lower bound) and *p* = 0.221 (upper bound) for the good performers. Despite the mean deviations not being statistically significant from 0, statistical equivalence could hence not be confirmed. When applying a more liberal equivalence interval of ± 4 semitones, however, TOST analyses confirmed statistical equivalence between the pitch matches and the Greenwood function for both the full group and the subgroup of good performers (*p* < 0.001 for lower bound in both cases; upper bound all: *p* < 0.001; upper bound good performers: *p* = 0.015). This indicates that, across subjects, the perceived pitch typically fell within a range of less than one octave around the Greenwood prediction.

**Figure 7:**
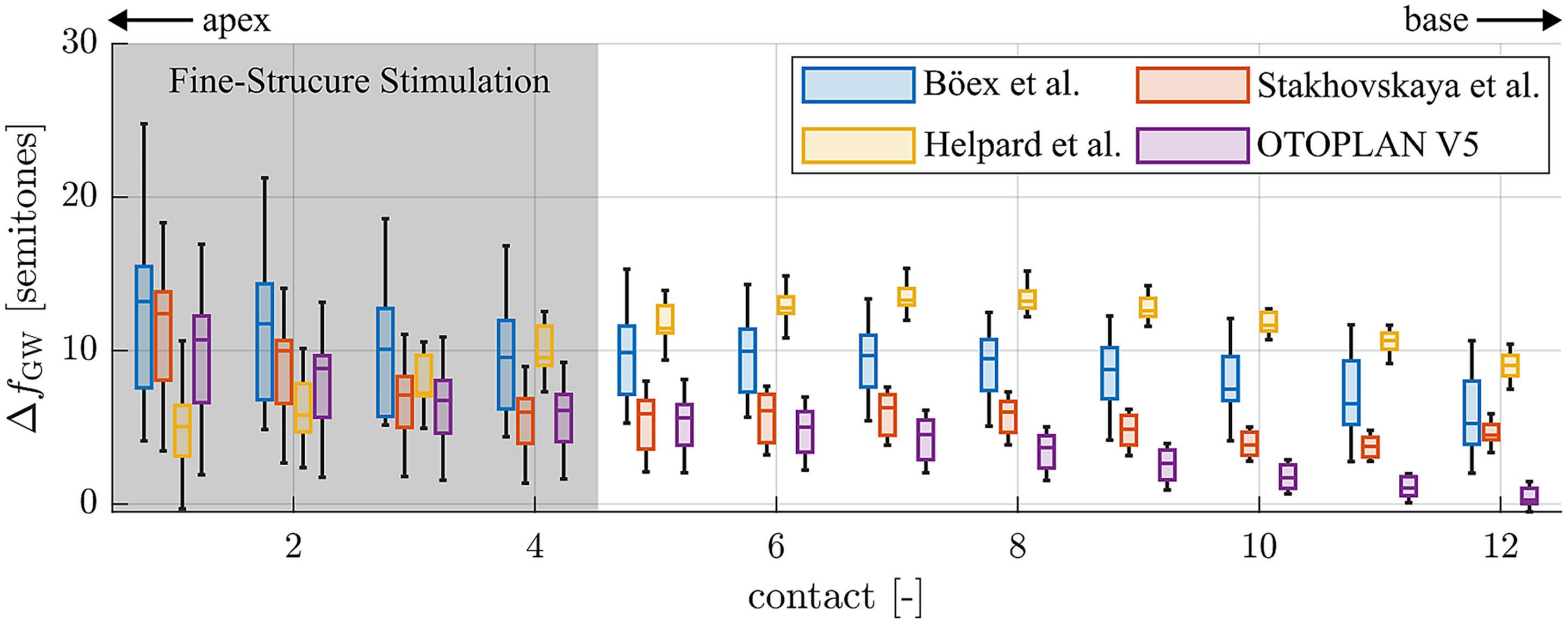
Deviations of tonotopic frequencies assigned to the single electrode contacts 1 (apical) to 12 (basal) when comparing different frequency allocation methods to the Greenwood reference proposed within this study.

In order to be able to compare the present study results to previous ones and identify potential reasons for different study outcomes, the tonotopic frequency assigned to each electrode contact of each one of the 12 study subjects was additionally computed with previously applied methods. This included the approaches proposed by Boëx et al. (2006), Stakhovskaya et al. (2007), Greenwood (1990) as well as values automatically calculated with the most recent release of the surgical planning platform OTOPLAN® (version 5, CAScination AG, Switzerland). The latter employs the previously proposed ECA method (Schurzig et al., 2018b) for computing the relative length *x* of the BM along the cochlear angle, which is then converted into frequency using the Greenwood equation (cf. Equation 1). The differences of frequency values computed with these methods to the Greenwood reference proposed within the present study were computed, the result of which is depicted in Figure 7. It is shown that all methods tend to estimate frequencies higher than suggested by the Greenwood reference, often reaching deviations of an octave or more. Furthermore, large discrepancies between approaches can be observed, and the range of deviations is largest for all methods within the apical region, i.e., where fine-structure stimulation is employed in case of the present study subjects.

## 4 Discussion

The success of cochlear implantation is typically evaluated based on a patient’s postoperative speech perception, which is known to be affected by demographic factors, such as duration of hearing loss and age at implantation (Blamey et al., 2013), as well as cognitive and linguistic abilities (Collison et al., 2004). In addition, implantation-related factors such as sufficient coverage of the intracochlear neural structures by the inserted electrode array have been shown to positively influence speech perception outcomes (Breitsprecher et al., 2023; Weller et al., 2023). Furthermore, the resulting decrease of frequency mismatch between the natural, tonotopic frequency distribution of the inner ear and the frequency assignment of the CI array provides additional improvements in speech perception (Canfarotta et al., 2020) and sound quality (Vermeire et al., 2008; Reiss et al., 2007). A requirement for achieving sufficient coverage of the cochlea is the consideration of anatomical differences among patients; differences in cochlear size require different CI array lengths to achieve sufficient insertion angles and corresponding coverage of the intracochlear neurons (Timm et al., 2018; Mistrík and Jolly, 2016), and the distribution of the neurons themselves is dependent on the individual anatomy as well (Li et al., 2021; Helpard et al., 2021b). Based on these findings, the question arises if there are interindividual differences in the mapping of the audible frequency spectrum onto the intracochlear neurons as well, and if the consideration of these differences could yield further advances in pitch perception and sound quality with a CI.

Previous studies have been conducted on cochlear implant users with residual hearing on the contralateral side, typically with the goal to compare the frequency perception of these subjects to the Greenwood projection of the audible frequency range onto the BM (Greenwood, 1990). CI patients with contralateral residual hearing are uniquely well-suited for this task as they can provide comparative feedback on the auditory percept in response to CI stimulation while radiological imaging yields information on the place of the stimulus. Previous studies have confirmed that low frequency sounds are perceived apically in the cochlea while high frequency sounds are perceived further toward the base (Townshend et al., 1987; Dorman et al., 1990; Busby et al., 1994).

Unfortunately, different study outcomes were derived regarding correspondence to the Greenwood function. While some studies found good agreement of Greenwood’s formula with the pitch matches derived from SSD subjects (Vermeire et al., 2008; Adel et al., 2019), other studies found substantial deviations from Greenwood (in the range of several octaves toward the base, i.e., frequencies are perceived lower than suggested by Greenwood) (Boëx et al., 2006; McDermott et al., 2009; Zeng et al., 2014; Dorman et al., 2007; Peters et al., 2016). One factor contributing to these differences may be the type of implanted electrode array and the corresponding coverage of the intracochlear neural structures. Vermeire et al. as well as Adel et al. conducted their study on patients implanted with long electrode arrays (either 28 mm or 31.5 mm length) and accordingly high neural coverage. In contrast, the insertion depth values reported within the studies reporting large deviations to Greenwood were substantially shorter and partially pre-curved [e.g., 16–22 mm in (McDermott et al., 2009), 15–25 mm in (Boëx et al., 2006; Peters et al., 2016)]. The result of shallower insertions and or pre-curved arrays may be that with increasing duration of implant use, the auditory system adapts to the stimulation of a spatially more restricted area of the intracochlear neurons (McDermott et al., 2009; Reiss et al., 2007).

One aspect that most studies on pitch perception with a CI have in common is that many simplifying assumptions are applied when deriving the tonotopic frequencies assigned to single CI electrode contacts, i.e., in applying the Greenwood function in a clinical setting. As mentioned before, the function only maps the audible frequency range of 20–20,000 Hz onto the BM which is not visible in clinical CT, hence requiring additional models for clinical frequency allocation. As shown in Figure 7, not all models are well-suited for determining tonotopic frequencies of individual electrode contacts. The one proposed by Böex et al., for instance, consistently overestimates tonotopic frequencies by about one octave. The modelling assumptions alone hence explain a substantial part of the basal frequency shift reported by the authors. In addition to the mathematical simplifications, Boex et al. derive their Greenwood frequency projection exclusively based on postoperative Stenver’s view radiographs of CI patients (Boëx et al., 2006). Based on this single image which is acquired such that it creates a visual top-down representation of the electrode array (Marsh et al., 1992), the superior semicircular canal and vestibule are used as landmarks to approximate the location of the RW and successively, the angular insertion depths of the individual electrode contacts. The authors then assume that the electrode array is located right beneath the BM and used the specifications of the array and the average BM length of 35 mm (Greenwood) to approximate tonotopic contact frequencies. The same method was employed by many other studies on pitch perception in CI patients with contralateral residual hearing (Dorman et al., 2007; Zeng et al., 2014), although many factors are neglected which make this approach highly inaccurate. Firstly, electrode arrays are often not fully inserted (Avallone et al., 2021; Zeng et al., 2014), such that electrode array length and BM length cannot be directly related. Secondly, electrode arrays do not always lie right beneath the BM, either because they are designed to take a more perimodiolar position (Dhanasingh and Jolly, 2017; Pietsch et al., 2022) or because their flexibility makes them change their intracochlear path according to the spatial boundary conditions of the scala tympani (Schurzig et al., 2021; Avci et al., 2014; Salcher et al., 2021). Hence, the insertion depth of an electrode array does not directly correspond to the BM length covered by the array. Furthermore, it was shown in histologic studies (Stakhovskaya et al., 2007) as well as on very high-resolution synchrotron radiation phase-contrast imaging (Li et al., 2021; Helpard et al., 2021b) that a specific angular location along the cochlear spiral does not correspond to one specific frequency, but that the angle-to-frequency relation is dependent on the individual cochlear anatomy. All of these simplification assumptions likely account for at least part of the basal frequency shift of pitch matches reported in studies employing these methods.

In the present study, the most recent key findings in intracochlear anatomical interrelations, anatomical differences between subjects as well as the exact intracochlear path of the electrode array were considered when deriving the location of individual electrode contacts along the BM. Furthermore, only patients with fully inserted long flexible electrode arrays and fine-structure stimulation were included in the investigation, i.e., patients where most of the intracochlear neural structures are stimulated electrically by the array, and where not only spatial but also temporal information is delivered within the apical cochlear region. Limiting the study to this patient cohort reduces the degree of extrapolation of actual pitch matching results beyond the location of the array itself. Our results showed that for the investigated patient cohort, Greenwood’s function approximates the subjects’ subjective perception of pitch well, at least for the investigated frequency range of 100 Hz to 8 kHz (Figure 6) and for the employed FS4 stimulation strategy. However, TOST analysis showed that the 90% confidence intervals exceeded the upper equivalence bound of 1 semitone for both the full study cohort and the good performers only. Statistical equivalence could hence not be confirmed. However, the observed mean deviations were small and close to the equivalence threshold. Furthermore, when using the broader interval of ± 4 semitones, pitch matches for both the full group and the good performers were statistically equivalent to the Greenwood prediction. This suggests that, despite individual variability, the Greenwood function provides a reasonable approximation of perceived pitch in single-sided deaf CI users, particularly among good performers and independent of the used frequency assignments. The present data on subjectively perceived pitch therefore helps to explain why ABF, or the adjustment of frequency assignments to the Greenwood function, can yield previously reported improvements in postoperative audiological outcomes, including speech comprehension and the subjective impression of the sound quality (Kurz et al., 2022; Kurz et al., 2023; Dillon et al., 2023; Kurz et al., 2024). Furthermore, the derived results on Greenwood frequency determination (Figure 7) suggest that with the most recent release of OTOPLAN©, commercial solutions represent one of the most reliable approaches for applying ABF within the clinical routine.

Previous pitch matching studies reported subjectively perceived frequencies to be lower than suggested by Greenwood (Zeng et al., 2014; Boëx et al., 2006; McDermott et al., 2009). In contrast, the good performers of the present study cohort rated the perceived pitch between 500 Hz and 4 kHz to be slightly higher than suggested by Greenwood (Figure 5), although these deviations were not statistically significant. This difference in study outcomes may be owed to the previously mentioned assumptions applied in previous studies to assign tonotopic frequencies to electrode contacts. Lower frequency values were also reported for recent studies in which electrocochleography (eCochG) measurements were employed to generate tonotopicity maps of the cochlea (Walia et al., 2024a; Walia et al., 2024b). However, while it is desirable to employ objective measurements for the derivation of individualized, tonotopic maps of the cochlea, it is unclear if the responses measured in these studies truly corresponded to the pitch perceived by the study subjects. Rather, literature suggests that for a certain acoustic frequencies, the eCochG peaks at a location that is basal to the characteristic place of that frequency as per Greenwood (Cheatham et al., 2011), such that the approach of confirming or disproving the Greenwood function via eCochG seems questionable in itself.

Another factor which must be considered is the distribution and survival of the intracochlear neural structures which will likely influence subjective percepts in response to electrical stimulation (Zeng et al., 2014). Poor neural survival in specific cochlear regions (Garcia et al., 2021; Pfingst et al., 2015) may affect the clarity of perceived pitch with a CI and may therefore be the reason why some subjects in the present study perceived a very similar pitch in response to stimulation with different, neighboring contacts (e.g., the most basal, high frequency contacts of subj06 and subj10 in Figure 4). This could be owed to both contacts stimulating the remaining neurons susceptible to electric stimulation. Neural health should therefore be considered in addition to the natural cochlear tonotopicity when creating individualized frequency assignments, as inconsistent neural survival must be expected in CI patients and the logarithmic nature of the Greenwood function may not always be a suitable model to interpolate frequencies in between measured percepts for hearing impaired individuals.

Another factor that may have influenced the pitch matching results is the intensity of electrical stimulation. Although stimulus intensity was kept constant within subjects during testing, previous studies have shown that increases in current level can lead to shifts in perceived pitch, particularly in apical regions of the cochlea (e.g., Arnoldner et al., 2006). This effect is thought to arise from broader current spread or recruitment of more basal neural populations at higher intensities. While we did not systematically vary intensity across subjects or electrodes, this limitation should be considered when interpreting the variability in pitch matches—especially in cases where neighboring electrodes elicited similar percepts. Future studies may benefit from explicitly examining the interaction between intensity and pitch perception to refine individualized frequency mapping strategies.

## Data Availability

The raw data supporting the conclusions of this article will be made available by the authors, without undue reservation.
