# Supplementary material for: The Greenwood function shows close alignment with pitch perceived by cochlear implant patients with long, flexible electrode arrays and fine-structure stimulation
Source: Front Neurosci. 2025 Sep 2;19:1624499. doi: 10.3389/fnins.2025.1624499 (PMC12457772; doi:10.3389/fnins.2025.1624499)
Supplement: Supplementary file 1 [file Data_Sheet_1.PDF]

Supplementary material to:

The Greenwood Function Shows Close Alignment with Pitch Perceived by Cochlear Implant Patients with Long, Flexible Electrode Arrays and Fine-Structure Stimulation

Andreas Büchner[1,2*], Tobias Weller[1,2*], Richard Penninger[3], Luke Helpard[3], Hanif M. Ladak[4,5,6], Sumit Agrawal[4,5,6], Thomas Lenarz[1,2], Daniel Schurzig[1,3]

[1] Dept. of Otorhinolaryngology, Hannover Medical School, Hannover, Germany

[2] German Hearing Center (DHZ), Hannover, Germany

[3] MED-EL, Innsbruck, Austria

[4] Department of Otolaryngology-Head and Neck Surgery, Western University, London, ON, Canada

[5] Department of Medical Biophysics, Western University, London, ON, Canada

[6] Department of Electrical and Computer Engineering, Western University, London, ON, Canada

* Contributed equally to the study

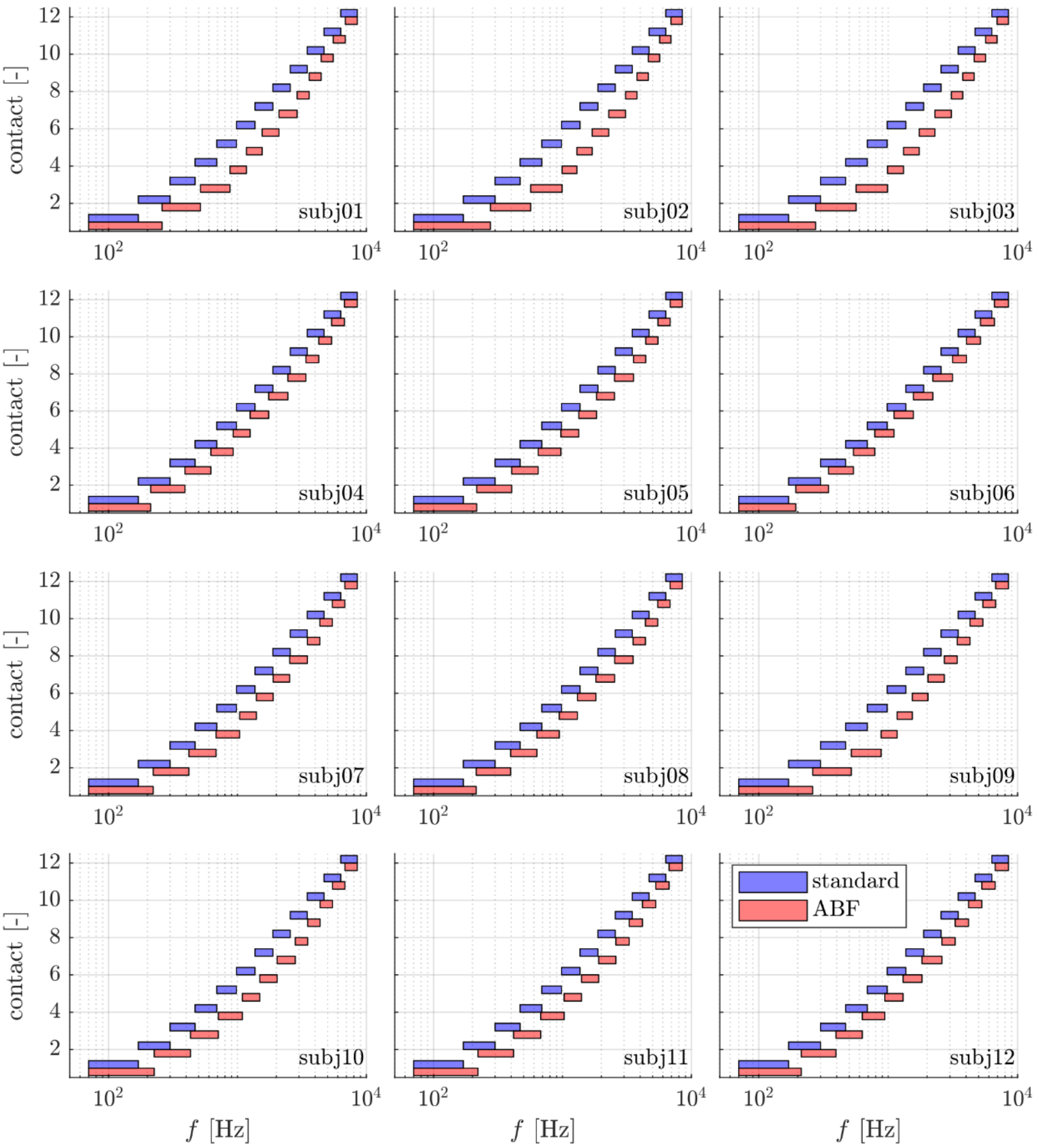

Figure S1: frequency band comparison of the subjects' standard and individualized ABF maps, respectively

frq range [Hz]		all	good performers
<250	N [-]	10	5
	p [-]	1.000	0.313
	median [semitones]	-1,2	-3,6
250-500	N [-]	11	4
	p [-]	0.638	0.620
	median [semitones]	-1,6	1,2
500-1k	N [-]	14	6
	p [-]	0.068	0.063
	median [semitones]	4,8	4,8
1k-2k	N [-]	13	5
	p [-]	0.685	0.125
	median [semitones]	0,7	2,4
2k-4k	N [-]	12	5
	p [-]	0.176	0.188
	median [semitones]	2,4	4,1
>4k	N [-]	12	5
	p [-]	0,910	1.000
	median [semitones]	1,2	0,8

Table S1: results of the Wilcoxon Signed Rank test for the differences of the pooled average pitch matching frequencies and the respective Greenwood frequency values depicted in Fig. 4
